# Safety and efficiency of a redirection procedure toward an out of hours general practice before admission to an emergency department, an observational study

**DOI:** 10.1186/s12873-018-0173-6

**Published:** 2018-08-22

**Authors:** Charles Morin, Jacques Choukroun, Jean-Christophe Callahan

**Affiliations:** 1 Service de Médecine Polyvalente, Centre Hospitalier du Mans, 194 Avenue Rubillard, 72000 Le Mans, France; 2 Service des Urgences Adultes, Centre Hospitalier du Mans, 194 Avenue rubillard, 72000 Le Mans, France; 3 Service de Réanimation Polyvalente, Centre Hospitalier du Mans, 194 Avenue Rubillard, 72000 Le Mans, France

**Keywords:** Emergency department management, Patient redirection, Patient safety, Primary care

## Abstract

**Background:**

Primary care patients are often cited as a cause of Emergency Department overcrowding (ED). The aim of this study was to evaluate a physician led redirection procedure of selected patients towards an out of hours general practice (OHGP) in an Emergency Department with 55,000 admissions per year.

**Methods:**

Observational monocentric study over a period of 2 months. Every patient redirected to the OHGP was included and subsequently contacted by telephone to answer a standardized questionnaire, in order to measure:

- Redirection rate over the entire period and during weekdays or weekends/holiday
- Rate of redirected patients who went to the OHGP
- Rate of redirected patients who consulted in an ED in the next 72 h for the same reason
- Redirected patients’ satisfaction rate

**Results:**

During the study period 9551 patients presented to the ED, of which 288 were redirected towards the OHGP (3%). The redirection rate was 1.9% during weekdays and 5.7% during weekends/holiday (*p* < 0.001). Of the redirected patients, 77% answered the telephone interview.

Ninety percent of these patients consulted the OHGP. The main reasons for not consulting were: unduly long wait, opening hours not suitable, too costly. The rate of redirected patients who consulted in an ED in the following 72 h for the same reason was 4.1%. The satisfaction rate was 79.6% among interviewed patients.

**Conclusions:**

A physician led procedure to redirect selected patients from the ED towards an OHGP results in a low redirection rate, unlikely to have a significant effect on ED patient flow. However, the procedure is safe and well accepted by a majority of patients.

## Background

Despite differences across health care systems, primary care patients treated in the emergency department (ED) are usually defined as lower acuity patients, unlikely to be admitted, and who could have been handled by a primary care provider outside of the ED [1]. In France, EDs can be accessed after telephonic referral by the Emergency Medical Service (SAMU), after referral by a General practitioner (GP), or by self-referral. In a one-day observational study conducted in every ED in France on the 11th of June 2013, out of 52,018 patients 58% were self-referrals and it was considered that 16.4% of all ED admissions could have been handled the same day in a primary care setting had there been the possibility [2].

Between 2002 and 2012, the number of admissions in EDs in France has increased by 30% (to 18 million admissions for a population of 65.8 million), and ED overcrowding has become a public safety issue [3]. Several international studies have found a correlation between ED admissions during a period of overcrowding and excess morbidity [4–6]. Admission of low acuity patients that could have been seen in a general practice setting, while not the only factor in ED overcrowding, can have a negative impact on patient flow by diverting resources needed elsewhere [7–9]. Furthermore, low acuity patients are more likely to leave the ED without being seen [10]. An ED admission for these patients is also more costly than a general practice consult from an economic standpoint [3, 11, 12].

In France, non-emergency unscheduled care can be handled during nights, weekends and holidays by general practice physicians in out of hours general practice clinics (OHGP). These clinics are not equipped for imaging or laboratory testing. Redirecting patients presenting to the ED with complaints not requiring the level of care of an ED towards an OHGP reduces the number of admissions, especially during periods of overcrowding. This practice exists in a majority of University Hospital EDs [13]. However, there exists potential risks with such a procedure by misestimating the severity of the clinical picture presented by the patient.

Therefore, the aim of this study was to assess whether such a procedure is effective, safe, and satisfactory for the patients.

## Methods

### Setting and study design

We conducted a prospective observational study. All patients presenting to the adult ED of the Le Mans general hospital between February first and March 31st 2016 were screened for inclusion.

The department averages 155 admissions per day. Every patient presenting to the ED is evaluated within minutes by a triage nurse according to a pragmatic standardized triage scale. The redirection procedure towards the OHGP is summarized in Table 1.

**Table 1:** Redirection procedure towards the OHGP

Redirection is warranted after medical validation by a senior emergency physician if the following criteria are met: - Absence of priority criteria according to the triage scale, - The patient was not referred to the ED by a physician, - The OHGP is easily accessible by the patient, - The patient is covered by social security, - Informed consent of the patient was obtained concerning the redirection. If the patient refuses the redirection he is admitted to the ED.	

A time stamped administrative record is created for every redirected patient. This record contains the patients age, sex, contact details and whether the patient has a declared primary care physician.

The OHGP is easily accessible on foot from the ED. It is open from 20 h00 to midnight Monday through Friday, from 12 h00 to midnight on Saturday, and from 08 h00 to midnight on Sunday and holidays.

During the study period, every adult patient redirected towards the OHGP was included in the study and received an information sheet explaining the aims of the study and a summary of the protocol. Redirected patients were contacted by telephone between 72 h and 15 days after their initial presentation to the ED in order to answer an 18 question semi-structured questionnaire. The aim of this questionnaire was to elicit answers concerning the primary and secondary complaints, patient pathway before and after the redirection, understanding and satisfaction of the redirection, whether further testing, specialist referrals or ED visits were necessary for the same complaints following the OHGP visit. Patient complaints were classified according to the International Classification of Primary Care (ICPC), developed by the World Organization of family Doctors (WONCA). Telephone interviews were conducted by a single investigator in order to limit the risk of bias.

A patient was considered as a non-responder if five calls went unanswered between 72 h and 15 days after inclusion.

### Outcome measures

Redirection rate was defined as the proportion of patients presenting to the ED who were subsequently redirected towards the OHGP during the study period. A secondary analysis was performed comparing the redirection rates during weekdays and during weekends or holidays. We also sought to determine the reason why a primary care physician was not consulted instead of coming to the ED.

Safety was evaluated using the rate of patients redirected towards the OHGP who were subsequently admitted in an ED in the next 72 h for the same complaint. We also analyzed the complaints, final diagnostic and treatment pathway for these patients.

Accessibility rate was defined as the proportion of redirected patients who actually consulted the OHGP. In case of non-consultation, the reason was requested.

Satisfaction was measured by the proportion of redirected patients who felt that the redirection was justified according to their primary complaint. In case of non-satisfaction, the cause was ascertained.

### Data analysis

Continuous variables were presented as means and standard deviation; categorical variables were presented as absolute values and percentages. Proportions were compared with the Chi2 test using R software. Tests were bilateral with a type 1 error defined at 5%.

Using a power calculation with a type 1 error of 5%, an absolute precision of 4%, and an estimated maximum rate of readmitted patients at 72 h of 10% we calculated that at least 216 patients were necessary for analysis. In order to ascertain the generalizability of the telephonic interview results with the entire reoriented population we compared these two groups according to demographic data, whether the reorientation was performed during a weekday or not, during daytime (08 h00 to 20 h00) or not, and whether a treating physician was declared. The only difference between variables to reach statistical significance was the proportion of patients having a declared treating physician (77.6% in the non-responder group vs. 88.7% in the responder group, *p* = 0.02).

## Results

### Patient characteristics and redirection rate

Demographic data are summarized in Table 2. Between February 1st and March 31st 2016, 9551 patients presented to our ED. Of these, 288 were redirected towards the OHGP (overall redirection rate 3%). Redirection rate was 5.7% during week-ends and holidays, against 1.9% during weekdays (*p* < 0.001).

**Table 2:** Patient demographics. SD: standard deviation

	All patients	Redirected patients
Total:	9551 (100%)	288 (3%)
Weekdays	6815 (100%)	132 (1.9%)
Week-ends and holidays	2736 (100%)	156 (5.7%)
Gender:		
Male	4897 (51.3%)	131 (45.5%)
Female	4654 (48.7%)	157 (54.5%)
Age (SD)	53 (27.7)	36 (15.4)

86% of redirected patients declared having a corresponding primary care physician.

### Characteristics of responders

Two hundred and twenty one out of 288 reoriented patients answered the telephonic interview (76.7%).

A majority of responders (178; 80.5%) did not try to contact their GP before presenting to the ED. For 118 of these patients (53.4%) their usual GP was absent or the practice was closed. For 194 patients (87.8%), had their GP been able to see them the same day they would have chosen that option.

The most frequent primary complaints are summarized in Table 3.

**Table 3:** Ten most common primary complaints among redirected patients

Primary complaint	Number of patients (%)
Hyperthermia	24 (10.9%)
Lumbar symptoms	23 (10.4%)
Dental or gingival symptoms	18 (8.1%)
Throat symptoms	15 (6.8%)
Dysuria	10 (4.5%)
Localized rash	10 (4.5%)
Cough	9 (4.1%)
Lower limb symptoms	8 (3.6%)
Abdominal pain	7 (3.2%)
Ear pain	7 (3.2%)

### Safety

Nine redirected patients were admitted to an ED in the following 72 h for the initial complaint (4.1%).

One of these patients had to be hospitalized because of an anal abscess. It was the only patient redirected to the ED from the OHGP.

### Accessibility

One hundred and ninety nine reoriented patients consulted the OHGP (accessibility rate 90%). The main reasons for not presenting to the OHGP were: excessive waiting times (9 patients), opening hours not suitable (5 patients) and consultation costs (4 patients).

Twenty nine percent of redirected patients had never heard of the OHGP before presenting to the ED.

### Patient satisfaction

For 176 patients the redirection from the ED was justified according to their primary complaint (satisfaction rate 79.6%). The main causes for non-satisfaction were the absence of imaging modalities or laboratory testing in the OHGP (12 patients), absence of specialist (including dentist) consultation (8 patients), pain too important (4 patients), “should have been seen in the ED” (4 patients), and excessive waiting times (4 patients).

## Discussion

The results of our study show that relatively few patients are redirected from the ED towards an OHGP, although the redirection rate is higher during week-ends and holidays. The redirection procedure is safe, with few patients requiring a follow-up in an ED in the 72 h post-redirection, and without prejudice for their level of care. Furthermore, a majority of patients believed that their redirection from the ED was justified.

Few studies have evaluated in different health care systems to what extent patients with minor ailments can be redirected from the ED towards a primary care provider. Using a redirection procedure similar to ours, Bentley found a higher redirection rate of 7% in a Scottish teaching hospital, with a sub-optimal care rate of 0.23% as a result of the redirection [14]. In our study, only one of the 221 redirected patients who could be contacted for the interview required a subsequent hospitalization, for a benign condition.

Van der Straten evaluated the use of the Manchester Triage Scale by trained nurses at an “Acute Care Post” to separate flows of patients between an ED and an OHGP [15]. Seventy one percent of patients were directed towards the GP, and out of the sub group of less urgent patients initially oriented to the GP, 6.5% were redirected towards the ED with a 1.2% hospitalization rate in the 4 weeks following the initial triage. The large difference in the triage rate towards the GP between this study and both the Scottish and our study may be explained that triage was done at an earlier stage, before ED presentation. In France and Scotland this is done by centralized telephone advice and triage services (SAMU / Centre 15 for France and NHS 24 for Scotland). Despite these differences, all three studies demonstrate that it is possible to safely redirect low acuity patients to a GP before the ED.

The low redirection rate in our study is unlikely to have a significant impact on ED flow and does not support the idea that primary care patients are a major burden on EDs. However, in the French 1 day cross-sectional study of 2013 [2], it was considered that 16.4% of all ED admissions could have been handled the same day in a primary care setting had there been the possibility. This appraisal was done at discharge from the ED by the treating emergency doctor, and thus is subject to hindsight bias. Nerveless, it raises the question whether our redirection procedure might be too selective and whether more patients could be redirected in our ED. In a systematic review, Durand found that the proportion of low-acuity ED visits ranged from 4.8 to 90% depending on studies [16]. This variability could be explained by different health care systems, the lack of consensus for defining low-acuity ED visits and also whether the categorization was done prospectively at triage or retrospectively. Additionally, there is a distinction between a low-acuity ED visit and a patient that can be safely and appropriately redirected towards a less urgent primary care facility in a timely manner. Many patients require ED care not because of the severity of their symptoms but because of the technical aspects of their care which can be managed in an ED.

In our study the redirection rate was significantly higher during week-ends and holidays than during weekdays. This could be explained either by the longer opening hours of the OHGP during week-ends or by a higher rate of low-acuity complaints among patients presenting to the ED due to the diminished accessibility of GPs during these periods. An argument in favor of this second hypothesis is that a majority of redirected patients in our study did not try to contact their primary GP because they believed he or she would be absent. Effects of an extended access to primary care facilities on ED visits are debated in the literature, with conflicting results. Whittaker found a correlation between the implementation of an extended access primary care service and a reduction of self-referring low-acuity patients by 26.4% in Manchester (United Kingdom) in 2014 [17]. In a study by Martsolf however, during the period between 2007 and 2012 in 23 American states, no association was found between the opening of a retail clinic and ED visits for low-acuity patients [18]. Despite differences in setting and methods, these studies underline the complexity of health care systems and their responses to heterogeneous primary care demands.

Our study shows that redirecting lower acuity patients toward an OHGP is unlikely to affect ED patient flow due to the low redirection rate. Despite this low redirection rate, we believe that this procedure still has its usefulness. ED and GP services should not be considered interchangeable. Each has its own specificities, and redirection should be considered as a service aimed at giving the patient the most adequate care, not as a means to lower ED flow.

In our study, 10% of redirected patients did not follow the instruction to go to the OHGP. Although none of these patients presented an unfavorable outcome, they present a challenge in the sense that not all redirected patients have benign self-limiting conditions. Furthermore, not requiring an ED consult does not mean that no medical advice is warranted. In particular, ED doctors should remember to enquire whether the patient has the ability to pay in advance the fee for the OHGP.

## Limitations

This study has several limitations. As an observational study, it was not designed to measure a possible impact of a redirection procedure on traditional measures of patient flow in the ED such as mean length of stay. As a single center study, the results should not be directly generalizable to EDs in different health care systems. As there was no change in the redirection procedure, no internal comparison was possible. The study was also open to selection bias, as different care providers at triage might have different assessments of what is a complaint requiring a GP. It is possible that a certain number of patients were admitted to the ED who could have benefited from the procedure and the study was not designed to measure this. Also the number of patients who refused redirection was not measured. The data for this study was collected in February and March and any seasonal variations in pathologies that could influence the redirection rate are not accounted for. Despite the standardized nature of the telephone interview, it is subject to recall bias. For example, patient’s initial feeling about the redirection might have a different opinion of the procedure after a few days depending on the evolution of their ailment. Almost a quarter of redirected patients did not respond to the telephonic interview. However, comparison of demographic data between the group of responders and non-responders did not find any significative difference, aside from whether there was a declared primary care physician.

## Conclusion

In summary, a physician led procedure to redirect selected patients from the ED towards an OHGP results in a low redirection rate, unlikely to have a significant effect on ED patient flow. However, the procedure is safe with few patients requiring a follow-up in an ED in the 72 h post-redirection, and without prejudice for their level of care. Furthermore, a majority of patients believed that their redirection from the ED was justified.

## Abbreviations

ED: Emergency Department
GP: General Practitioner
ICPC: International Classification of Primary Care
NHS: National Health Service
OHGP: Out of Hours General Practice Clinic
SAMU: Emergency Medical Service (Service d’Aide Médicale Urgente)
WONCA: World Organization of Family Doctors
